# Modulation of human postprandial lipemia by changing ratios of polyunsaturated to saturated (P/S) fatty acid content of blended dietary fats: a cross-over design with repeated measures

**DOI:** 10.1186/1475-2891-12-122

**Published:** 2013-08-16

**Authors:** Tilakavati Karupaiah, Kalyana Sundram

**Affiliations:** 1 National University of Malaysia, Bangi, Malaysia; 2 Faculty of Health Sciences, National University of Malaysia, Kuala Lumpur, Malaysia

**Keywords:** Postprandial lipemia, Dietary fats, P/S ratio, Lipoproteins

## Abstract

**Background:**

Postprandial lipemia (PL) contributes to coronary artery disease. The fatty acid composition of dietary fats is potentially a modifiable factor in modulating PL response.

**Methods:**

This human postprandial study evaluated 3 edible fat blends with differing polyunsaturated to saturated fatty acids (P/S) ratios (POL = 0.27, AHA = 1.00, PCAN = 1.32). A cross-over design included mildly hypercholestrolemic subjects (9 men and 6 women) preconditioned on test diets fats at 31% energy for 7 days prior to the postprandial challenge on the 8th day with 50 g test fat. Plasma lipids and lipoproteins were monitored at 0, 1.5, 3.5, 5.5 and 7 hr.

**Results:**

Plasma triacylglycerol (TAG) concentrations in response to POL, AHA or PCAN meals were not significant for time x test meal interactions (*P* > 0.05) despite an observed trend (POL > AHA > PCAN). TAG area-under-the-curve (AUC) increased by 22.58% after POL and 7.63% after PCAN compared to AHA treatments (*P* > 0.05). Plasma total cholesterol (TC) response was not significant between meals (*P* > 0.05). Varying P/S ratios of test meals significantly altered prandial high density lipoprotein-cholesterol (HDL-C) concentrations (*P* < 0.001) which increased with decreasing P/S ratio (POL > AHA > PCAN). Paired comparisons was significant between POL *vs* PCAN (*P* = 0.009) but not with AHA or between AHA *vs* PCAN (*P* > 0.05). A significantly higher HDL-C AUC for POL *vs* AHA (*P* = 0.015) and PCAN (*P* = 0.001) was observed. HDL-C AUC increased for POL by 25.38% and 16.0% compared to PCAN and AHA respectively. Plasma low density lipoprotein-cholesterol (LDL-C) concentrations was significant (*P* = 0.005) between meals and significantly lowest after POL meal compared to PCAN (*P* = 0.004) and AHA (*P* > 0.05) but not between AHA *vs* PCAN (*P* > 0.05). AUC for LDL-C was not significant between diets (*P* > 0.05). Palmitic (C16:0), oleic (C18:1), linoleic (C18:2) and linolenic (C18:3) acids in TAGs and cholesteryl esters were significantly modulated by meal source (P < 0.05).

**Conclusions:**

P/S ratio of dietary fats significantly affected prandial HDL-C levels without affecting lipemia.

## Background

Plasma triacylglycerol (TAG) in the post-meal state mainly comprises triacylglycerol-rich lipoproteins (TRL) carried by chylomicrons [1]. With human eating patterns reflecting a postprandial state in excess of 16 h per day, the fed state represents a major portion of this metabolic transition. Elevated TAG triggers a chain of metabolic reactions that may reduce high density lipoprotein-cholesterol (HDL-C), promote formation of small dense low density lipoprotein (LDL) particles and activate prothrombotic factor FVII [2-4]. The induction of endothelial dysfunction as well as a prothrombic state may contribute to cardiovascular dysfunction [5,6]. Thus the post-meal state, its resulting postprandial lipemia and increased generation of TRL are recognized as modifiable cardiovascular disease (CVD) risk factors [7]. In fact prolonged circulation of TRL and suppression of HDL-C concentrations are independently linked to atherogenicity progression and early death in type 2 diabetes with similar associations observed in metabolic syndrome, obesity and hypertriglyceridemia [8,9].

Current dietary fatty acid recommendations in the Adult Treatment Panel (ATP) III guidelines focus on reduction of saturated fatty acids (SFA) to <7% en, increasing monounsaturated fatty acids (MUFA) as a replacement for SFA, maintaining polyunsaturated fatty acids (PUFA) at 10% en and reduction or elimination of trans fatty acids (TFA) [7,10]. TFA link to CVD has resulted in actions to remove TFA from the food supply chain. Their replacement options include substituting partially hydrogenated fatty acids with SFA from natural fat(s) or specifically designed fully hydrogenated interesterified fat containing SFAs made up mostly of stearic acid [11-14]. The ideal fatty acid replacement is postulated to reduce cardiovascular risk factors compared to TFA. A consensus opinion examining these options recommended further interventional and observational studies to fully understand the tradeoffs inherent in the proposed recommendation [15]. Recommendations supporting the reduction of SFAs to reduce coronary heart disease (CHD) risk are now been questioned on the basis that insufficient evidence exists to judge the effect on CHD risk of replacing SFAs with MUFAs [16]. Additionally many clinical trials evaluate humans in the fasted state but reports on the postprandial lipemic state is comparatively few. The effect of diet on a single biomarker such as lowering low density lipoprotein-cholesterol (LDL-C) is now considered insufficient evidence to assess CHD risk [17]. The combination of multiple biomarkers and the use of clinical endpoints are deemed useful to elucidate the mechanistic effects of diet on CHD.

Given CVD risk associations with enhanced lipemia, most initial research has targeted ‘fat load’ or ‘fat tolerance’ tests to differentiate responses between healthy subjects and those with metabolic abnormalities [1,2]. However the effect of individual dietary fatty acids on postprandial lipid metabolism remains to be fully addressed given the diversity of dietary fats and oils available for human consumption. Evaluation of physicochemical properties of oral fat meals suggests their fatty acid composition determines chylomicron size post-meal, which affects the rates of digestion and absorption [18]. This ultimately characterizes postprandial lipid response. Attenuation of prandial TAG responses has been shown with diets rich in n-3 PUFAs [19]. Studies using single fat meals to produce different SFA, MUFA and n-6 PUFA-rich diets report either a non-differentiated lipemic behavior or exacerbated or reduced TAG responses [19-21]. A recent study in making comparisons between SFA classes using palmitic-rich, stearic-rich or lauric + myristic-rich fats, found that slower prandial fat clearance was associated with increasing chain length of the saturates [22]. The newer interesterified fats with stereospecific repositioning of fatty acids on TAG structures have also been investigated postprandially [23].

What is the effect of a single meal that is rich in either n-6 PUFA or MUFA compared to a SFA-rich meal as a modifiable factor for eliciting a lesser prandial response and a faster return to the fasting state? To date postprandial studies comparing either the influence of a high-PUFA diet or a high-MUFA diet on lipemia are limited. The object of this study therefore was to evaluate single fat challenges of varying fatty acid polyunsaturated/saturated (P/S) ratios incorporated into test-meal challenges on postprandial lipoprotein and TAG responses in normal to mildly hypercholesterolemic humans.

## Methods

### Protocol

This study protocol was approved by the institutional ethics committee of the National University of Malaysia. Subjects were staff and students of a teacher training centre in Kuala Lumpur, Malaysia. Both normocholesterolemic (n = 9) and mildly hypercholesterolemic (n = 6) subjects without a history of atherosclerotic disease or hypertension were recruited. They were all non-smokers and did not consume any alcohol. None were on any prescribed medication, nutritional supplementation or weight-loss programs. Additionally, female subjects were not on oral contraceptives. Subjects were thoroughly briefed on the study protocol and gave their signed informed consent for participation in the study. They had the freedom to drop out from the study at anytime. Subject demographics were (mean ± SD): age, 35.5 ± 4.7 y ( range: 29 to 40 y); body mass index, 21.8 ± 2.3 kg/m^2^ (range: 17.3 to 26.7 kg/m^2^); total cholesterol, 5.26 ± 0.78 mmol/L (range: 3.97 to 6.90 mmol/L); triacylglycerol, 1.15 ± 0.60 mmol/L (range: 0.42 to 2.72 mmol/L); LDL-cholesterol, 3.09 ± 0.62 mmol/L (range: 2.60 to 4.72 mmol/L); and HDL-cholesterol, 1.64 ± 0.27 mmol/L (range: 1.32 to 2.20 mmol/L) [Table 1].

**Table 1:** **Subject characteristics (*n*** **=** ***15*)**

**Characteristics**	**Mean values ± sd**
Age (y)	35.5 ± 4.7
Sex	
*Male*	9
*Female*	6
Weight (kg)	56.6 ± 12.8
BMI (kg/m^2^)	
*All*	21.8 ± 2.30
*Male*	22.27 ± 2.93
*Female*	20.99 ± 2.16
% Body fat	
*All*	23.54 ± 6.53
*Male*	18.85 ± 6.27
*Female*	28.05 ± 4.70
Plasma total cholesterol (mmol/L)	5.26 ± 0.78
Plasma triacylglycerol (mmol/L)	1.15 ± 0.60
Plasma LDL cholesterol (mmol/L)	3.09 ± 0.62
Plasma HDL cholesterol (mmol/L)	1.64 ± 0.27

### Study design

The study was designed to evaluate the postprandial effects of 3 dietary fatty acid permutations differing in their PUFA and SFA content (P/S ratios 0.27, 1.00 and 1.32). Subjects were randomly assigned into 2 groups (Group A with 5 men and 3 women and Group B with 4 men and 3 women) which alternated between 3 diet rotations in a cross-over design. Prandial testing and assignment into groups for women was scheduled according to their menstrual cycle.

Breakfast, lunch and high tea meals were provided from a standard menu for the 7-day period leading to each postprandial investigation. This standardized menu plan was repeated for all 3 test fat rotations, which each group underwent with all meals prepared by a trained caterer and supervised by a dietitian. Portion size and protocols for incorporation of the test oils into meals preparation were fixed. The menu utilized typical Malaysian recipes and was constructed according to the following meal plan: *1)* for breakfast, a cereal dish and a snack item cooked with the test fat was served with either coffee or tea, *2)* lunch included fish or chicken and two vegetables cooked with the test fat and accompanied by rice and fruits and *3)* for high tea, a snack item with the test fat incorporated was served with either plain tea or coffee. A sample day’s menu for example provided fried noodles and doughnut for breakfast, followed by sweet and sour fish, spicy fried egg plant and stir fried green vegetables served with rice for lunch whilst subjects consumed cake and fried spring rolls for high tea. To maximize compliance, volunteers were provided with the test oils for preparing dinner as well as weekend meals at home. This 7-day period of dietary standardization minimized any variation in dietary fatty acid consumption before the postprandial investigations. A wash-out period of one week was allowed between the test rotations. Subjects were asked to eat according to their individual caloric plan as calculated by the dietitian. Body weight measurements were recorded before each postprandial challenge to ensure weight fluctuations were minimized between test meal rotations.

### Test diets and test oils

The P/S ratios of the diets were constructed by using palm olein in varying concentrations with other natural edible oils. The low P/S or POL diet (P/S = 0.27) was derived wholly from palm olein. This was compared to the American Heart Association-Step 1 or AHA diet (P/S = 1.0; palm olein and soybean oil blend) and a high P/S monounsaturated oil blend or PCAN diet (P/S = 1.32; palm olein and canola oil blend). The daily menu during the 7-day run-in period provided approximately 50g of the test fat in the diet equivalent to ~26% en. Thus total daily fat content of test meals provided during each of these periods was maintained at 31% en with the non-test fat contribution (~5% en) coming from dietary sources of invisible fats. All diets were eucaloric and only differed in their P/S ratios as demonstrated by the fatty acid composition of the test oils used for preparing the meals and actual analysis of the double portioned menus (Table 2).

**Table 2:** **Fatty acid composition of the test fats and test meals**^***1***^

**Fatty acid**	**Test Oils (% total fat**^***1***^**)**			**Test Diets (% total en**^***2***^**)**		
	**POL**	**AHA**	**PCAN**	**POL**	**AHA**	**PCAN**
**C12:0**	0.25	0.11	nd	nd	0.12	0.1
**C14:0**	0.84	0.39	0.21	nd	0.45	0.38
**C16:0**	37.07	20.94	10.56	38.62	22.01	13.49
**C16:1n7**	0.08	0.07	nd	nd	0.05	0.07
**C18:0**	3.94	3.75	2.07	3.7	3.92	2.92
**C18:1**	44.86	40.1	58.09	46.08	40.42	55.22
**C18:2**	11.03	27.93	18.77	11.6	26.15	18.07
**C18:3**	0.23	4.18	6.47	nd	3.65	5.1
*SFA*	*42*.*22*	*25*.*88*	*14*.*05*	*42*.*32*	*27*.*16*	*17*.*89*
*PUFA*	*11*.*38*	*32*.*25*	*25*.*5*	*11*.*6*	*30*.*01*	*23*.*6*
*MUFA*	*45*.*1*	*40*.*29*	*58*.*27*	*46*.*08*	*41*.*15*	*56*.*28*
**P/S**	0.27	1.25	1.81	0.27	1.10	1.32

^***1***^ values reported as percent of total fat in the meal.

^***2***^ % total en = as percent energy of total fat in the meal.

*POL* Palm olein, *AHA* Soybean-Palm olein blend, *PCAN* Rapeseed-Palm olein blend, *nd* not detectable, *SFA* saturated fatty acid, *MUFA* monounsaturated fatty acid, *PUFA* polyunsaturated fatty acid, *nd* not detectable, *P*/*S ratio* polyunsaturated/saturated fatty acid ratio.

### Postprandial challenge

Following the 7-day preconditioning, volunteers reported to the laboratory on the morning of the 8th day after an overnight fast of 10 h. A 12-ml fasted venous blood sample was obtained from the subjects. Subjects then completed consumption of a standard breakfast meal containing 50g of the test oil within 15 minutes of their baseline bleed. This meal consisted of 275 g of fried rice with 2 portions of breaded snack which provided approximately 1010 kcal, 101 g carbohydrate (40% en), 53 g fat (47% en) and 32g protein (13% en) (Table 3). Plain tea and coffee was allowed with the test meal. Subjects remained rested and in a fasted state throughout the 7-h postprandial period with blood drawing by venous puncture performed sequentially at 1.5, 3.5, 5.5 and 7.0 h after the fat challenge. The same sequence of blood sampling for all timed events was followed during each postprandial challenge. Blood sampling at each time point was completed for all subjects within 15 min of beginning the session. Mineral water consumption was permitted *ad libitum* throughout this period.

**Table 3:** Analyzed nutrient content of postprandial meal

**Nutrient content**	**Means +/- SD**
Total Energy (kcal)	1010 ± 250
Fat (g)	53.08 ± 16.25
% Fat energy	47.13 ± 7.29
Protein (g)	32.03 ± 11.00
% Protein energy	12.74 ± 3.68
Carbohydrate (g)	101.01 ± 30.15
% Carbohydrate energy	40.13 ± 6.03

*Note*- Energy value based on bomb calorimetry.

Macronutrient values based on proximate analysis.

### Analytical methods

#### Blood collection

Blood was collected into Vaccutainer® tubes (Becton Dickinson Vacutainer, Franklin Lakes, NJ, USA) containing EDTA (0.117 ml of 15% EDTA) and immediately centrifuged at 3000 × g for 20 min at 4°C (Sigma 3K12 B. Braun, Tuttlingen, Germany) to separate the plasma from red blood cells. About 3.0 ml of fresh plasma was reserved for ultracentrifugation whilst the remaining plasma was aliquoted and snap-frozen in liquid nitrogen and stored at −80°C for subsequent analyses.

#### Chylomicron separation

Ultracentrifugation of fresh EDTA plasma to separate the upper fraction containing triacylglycerol-rich lipoproteins (TRL) and HDL-C-rich bottom fractions was carried out in sealed Beckman Quick-Seal® polyallomer tubes (Beckman Instruments Inc., Palo Alto, CA, USA). Our laboratory technique for tube preparation and lipoprotein separation has been described elsewhere [22]. Three ml of fresh plasma was used and at the end of ultracentrifugation, tubes were sliced at the point of sealing, and aliquots removed in sequence. The bottom fraction was made up to a final volume of 3.0 ml with NaCl solution (*d* > 1.006 g/ml). This fraction was subsequently used to determine HDL-C concentration (*d* = 1.063 g/ml) as well as to characterize plasma cholesteryl ester (CE) FAC.

#### Lipid and Lipoprotein assays

TC in plasma and bottom fractions of ultracentrifuged plasma and plasma TAG were determined by enzymatic procedures [24,25]. HDL-C was precipitated with dextran sulfate- Mg^2+^ before assaying [26,27]. All assays were performed using a Cobas 6000 Chemistry Autoanalyzer System (Roche Analytic Instruments Inc, Nutley, NJ). Reagents, calibrators and controls were also supplied by the manufacturer (Roche Diagnostics Corporation, Indianapolis, IN). Plasma LDL-C was calculated by the differences between cholesterol content of the bottom fraction of ultracentrifuged plasma and HDL-C [28].

#### Determination of fatty acid composition (FAC)

Extracted lipids from TRL, CE and test meals were converted into fatty acid methyl esters before gas liquid chromatography for the determination of FAC (Perkin-Elmer Autosystem, Perkin-Elmer, Norwalk, CT, USA). The procedures have been described in detail elsewhere [22]. The FAC of the meals consumed by the subjects served to check whether the test meals achieved targeted FAC whilst the FAC of subjects’ plasma served to check compliance to the study protocol.

### Statistical analysis

The cross-over design enabled each subject to serve as his or her own control and all 15 subjects completed the 3 test meal rotations. The Statistical Package for Social Sciences, SPSS® for Windows™ application (Version 15.0, SPSS Inc., Chicago, IL., USA) was used for the required statistical analyses. Differences between outcomes from the various postprandial time intervals and baseline values (0 h) were interpreted as true measures of change resulting from dietary treatment. Multivariate analyses for repeated measures (MANOVA), using the general linear model (GLM), was performed for all time × test meal values for each measurement parameter. Univariate analysis was used to compare the area-under-the-curve (AUC) derived for the 7 h duration of the postprandial period calculated by the trapezoidal rule [29]. Levene’s Test was used to examine equality of variances across treatment groups. Bonferroni’s adjustment for multiple paired comparisons was used to test mean differences between treatment groups. Significance was set at *P* < 0.05 for all evaluated measures.

## Results

### Subject demographics

Mean (± SD) gain in body weight amounting to 0.97 ± 0.81 kg which was less than 2% of mean body weight at the start of the study was not significant [*data not shown*].

### Lipemic response and test fat clearance

**Figure 1:**
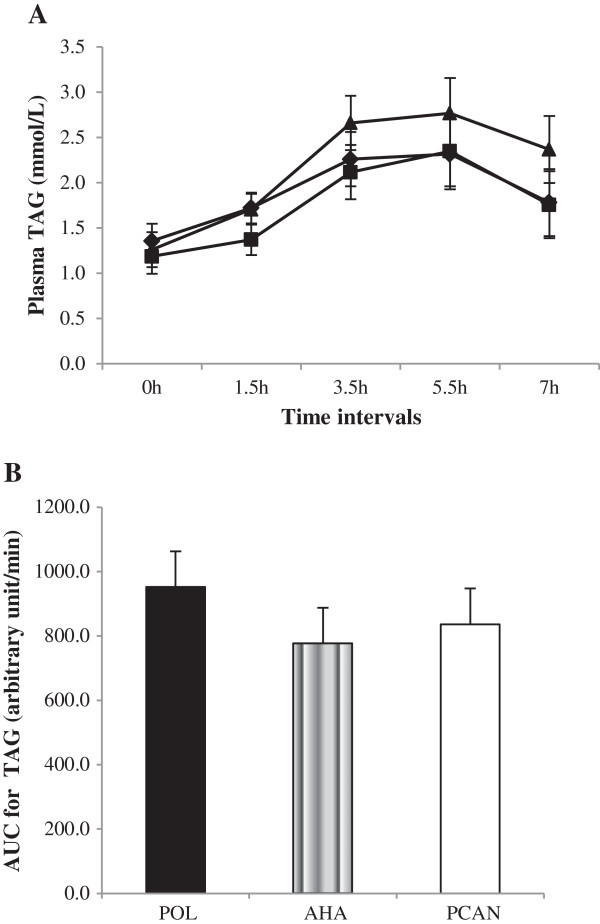
**Plasma TAG concentrations in response to test meals. A**: Plasma TAG (means ± SEM, n = 15) concentrations over 7 h in response to test meals containing POL, AHA or PCAN oil type. Repeated measures MANOVA analyzed for time x test meal interactions was not significant (*P* > 0.05) between treatments despite an observed trend (POL > AHA > PCAN). **B**: Univariate analyses for AUC data for plasma TAG trends was not significant between test meals (*P* > 0.05). In comparison AUC increased by 22.58% after the POL treatment and by 7.63% after the PCAN treatment compared to the AHA treatment. Legends **(A)**: POL = Palm olein only (); AHA = Soybean-Palm olein blend (); PCAN = Rapeseed-Palm olein blend ().

Based on postprandial plasma TAGs [Figure 1A], although a higher degree of lipemia was observed with decreasing P/S ratio (POL > AHA > PCAN) the effect of time × meal treatment changes was not significant (*P* > 0.05). TAG trends for all meals did not reach post-absorptive levels at 7h indicating fat clearance was not yet completed. AUC calculations indicated a lack of significance (*P* > 0.05) between meals [Figure 1B]. However TAG AUC increased by 22.58% after the POL meal and by 7.63% after the PCAN meal compared to the AHA meal (Table 4).

**Table 4:** Postprandial lipid and lipoprotein cholesterol concentrations in response to single meal fat challenges with varying P/S ratios

**Time**	**0 hr**	**1.5 hr**	**3.5 hr**	**5.5 hr**	**7 hr**	**AUC (arbitrary**	**POL *vs***	**POL *vs***	**AHA *vs***
**Diet**						**unit/min)**	**AHA**^**2**^	**PCAN**^**2**^	**PCAN**^**2**^
*Plasma Total Cholesterol (mmol/L)*^1^									
POL	5.58± 0.84	5.07 ± 0.81	5.32 ± 0.91	5.32 ± 0.83	5.43 ± 0.71	2224.38 ± 283.82	ns	ns	ns
AHA	5.76 ± 0.97	5.41 ± 1.19	5.49 ± 0.88	5.53 ± 0.98	5.39 ± 1.26	2310.38 ± 400.11			
PCAN	5.38 ± 1.21	5.25 ± 1.16	5.10 ± 1.03	5.51 ± 1.32	5.38 ± 1.36	2225.77 ± 482.45			
*Plasma HDL-C (mmol/L)*^1^									
POL	1.31 ± 0.31	1.57 ± 0.40	1.52 ± 0.29	1.54 ± 0.40	1.52 ± 0.36	635.61 ± 126.73	*P* = 0.005	*P* <0.001	ns
AHA	1.21 ± 0.37	1.34 ± 0.51	1.31 ± 0.35	1.21 ± 0.36	1.20 ± 0.37	533.93 ± 155.34			
PCAN	1.13 ± 0.33	1.16 ± 0.33	1.10 ± 0.31	1.12 ± 0.38	1.16 ± 0.37	474.30 ± 137.91			
*Plasma LDL-C (mmol/L)*^1^									
POL	3.69 ± 0.92	2.71 ± 0.77	2.58 ± 1.22	2.51 ± 1.12	2.82 ± 0.80	1258.63 ± 257.86	ns	*P* =0.004	ns
AHA	4.00 ± 0.86	3.45 ± 1.13	3.21 ± 0.87	3.24 ± 1.08	3.37 ± 1.23	1442.58 ± 354.17			
PCAN	3.63 ± 1.16	3.30 ± 0.97	2.96 ± 0.98	3.33 ± 1.18	3.41 ± 1.16	1374.91 ± 419.73			
*Plasma Triacylglyceride (mmol/L*) ^1^									
POL	1.26 ± 0.74	1.71 ± 0.67	2.66 ± 1.35	2.77 ± 1.81	2.37 ± 1.71	952.07 ± 496.11	ns	ns	ns
AHA	1.19 ± 0.60	1.37 ± 0.58	2.12 ± 1.13	2.35 ± 1.45	1.76 ± 1.25	776.67 ± 411.35			
PCAN	1.36 ± 0.86	1.72 ± 0.73	2.26 ± 0.99	2.32 ± 1.18	1.78 ± 1.30	835.91 ± 374.58			
*Plasma VLDL-C (mmol/L*) ^1^									
POL	0.49 ± 0.17	0.91 ± 0.18	0.82 ± 0.27	0.91 ± 0.15	1.05 ± 0.33	358.44 ± 54.56	ns	*P* =0.030	ns
AHA	0.48 ± 0.28	1.13 ± 0.38	0.95 ± 0.34	1.10 ± 0.35	1.32 ± 0.42	428.59 ± 79.40			
PCAN	0.51 ± 0.15	1.20 ± 0.35	1.14 ± 0.57	1.26 ± 0.56	1.36 ± 0.88	479.50 ± 165.90			

*POL* Palm olein, *AHA* Soybean-Palm olein blend, *PCAN* Rapeseed-Palm olein blend.

n = 15; ^1^means ± SD; ^2^Bonferroni’s pair-wise analysis for between diets comparisons; ns = *P* <0.05.

### Plasma TC, VLDL-C, LDL-C and HDL-C

Data for lipoprotein response as a result for diet is presented in Table 4. No change in time × meal treatment effects were observed in postprandial TC concentrations as a result of P/S ratio manipulation of the test meal challenges (*P* > 0.05).

**Figure 2:**
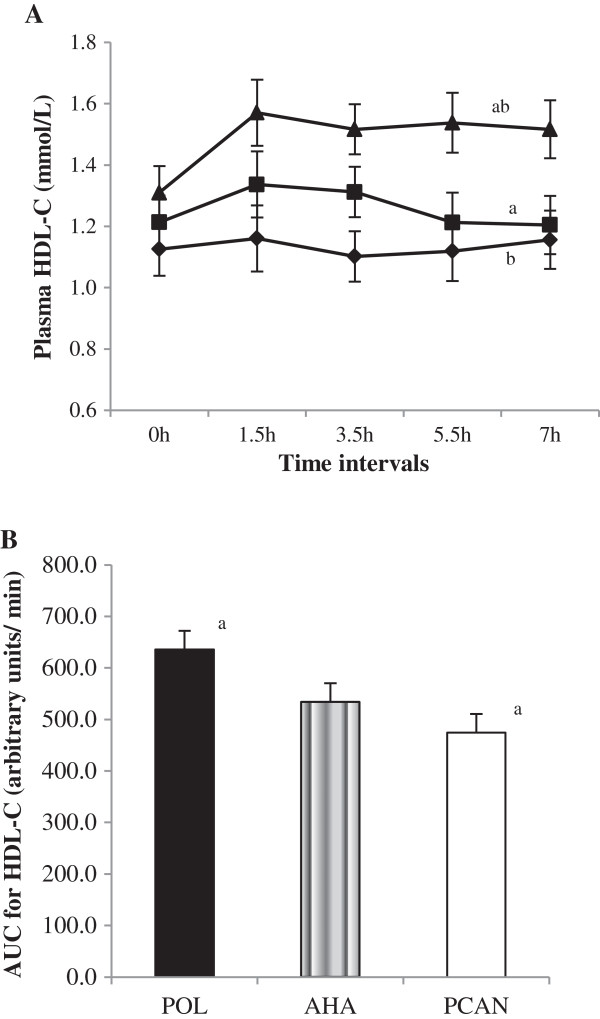
**Plasma HDL-C concentrations in response to test meals. A**: Plasma HDL-C (means ± SEM, n = 15) concentrations over 7 h in response to test meals containing POL, AHA or PCAN test fats. Varying the P/S ratios of test fat challenges significantly altered prandial plasma HDL-C concentrations (*P* < 0.001) when tested by repeated measures MANOVA analysis of time x meal interactions. Plasma HDL-C concentrations increased with decreasing P/S ratio (POL > AHA > PCAN). Bonferroni’s testing indicated significance for paired comparisons between POL with AHA (*a*, *P* = 0.005) and PCAN (*b*, *P* < 0.001) meals but not between AHA and PCAN (*P* > 0.05) meals. **B**: Univariate analysis indicated AUC for HDL-C AUC was greater after POL compared to PCAN (*a*, *P* = 0.009) and AHA (*P* > 0.05) meals. HDL-C AUC increased by 25.38% for POL compared to PCAN and 16.0% for AHA compared to PCAN meals. Legends **(A)**: POL = Palm olein only (); AHA = Soybean-Palm olein blend (); PCAN = Rapeseed-Palm olein blend ().

Varying the P/S ratios of meal challenges significantly altered (*P* < 0.001) prandial time × meal treatment changes in plasma HDL-C concentrations with an observed increasing trend of concentrations with decreasing P/S ratio (POL > AHA > PCAN) [Figure 2A]. The prandial increase for HDL-C was greater for POL compared to AHA (*P* = 0.005) and PCAN (*P* < 0.001) meals. This was substantiated by a significantly higher HDL-C AUC response for POL compared to AHA (*P* > 0.05) and PCAN (*P* = 0.009) meals which was measurably greater by 25.38% for POL compared to PCAN whilst AHA increased by 16.0% compared to POL [Figure 2B].

Postprandial LDL-C response in terms of time X meal treatment effects was significantly lowest after the POL treatment (*P* = 0.005) compared to AHA and PCAN with stronger differences between the POL and PCAN pair (*P* = 0.004) compared to POL and AHA (*P* > 0.05) or AHA and PCAN (*P* > 0.05) pairs. AUC for LDL-C remained unaffected by the P/S nature of the diets (*P* >0.05). A similarly lower trend for plasma VLDL-C (*P* = 0.032) for diet treatment × time interactions was observed with differences between POL compared to AHA (*P* > 0.05) and PCAN (*P* = 0.030). AUC for VLDL-C was affected by the P/S nature of the diets (*P* =0.017) and only mediated by the difference between POL and PCAN (*P* = 0.014).

### Fatty acid composition (FAC) of TRL and CE

**Figure 3:**
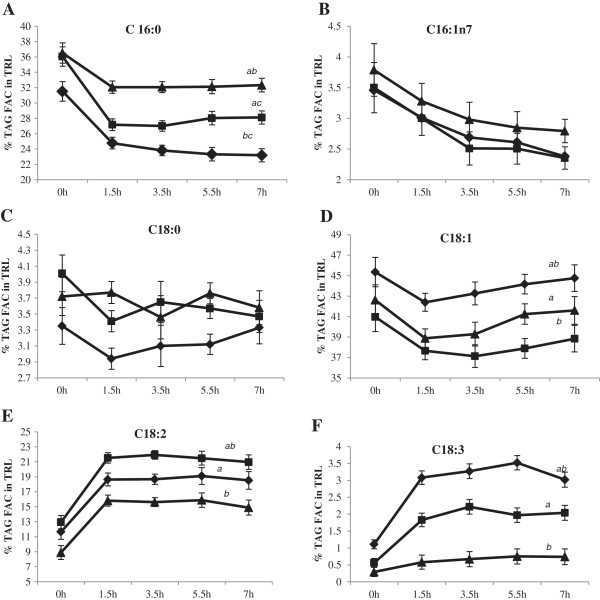
**Fatty acid composition (FAC) of triacylglycerol (TAG) in triacylglycerol-rich lipoproteins (TRL) (mean % ± SEM, n = 15) in response to test meals containing POL, AHA or PCAN test fats. **^*2*^Statistical analyses were corrected for baseline values and significance reported at *P* < 0.05 for time x meal interactions. **A**: Percent C16:0 trends after 1.5 h were significantly greater after the POL meal compared to AHA and PCAN meals (*ab*, *P* < 0.001), followed by the AHA meal and least after the PCAN meal (*c*, *P* = 0.029). **B**: Percent C16:1n7 incorporation into TRL TAG was not significantly different between treatments (*P* > 0.05). **C**: Percent C18:0 incorporation into TRL TAG was not significantly different between treatments (*P* > 0.05). **D**: Percent C18:1 significantly increased after the PCAN meal compared to POL (*a*, *P* = 0.001) and AHA (*b*, *P* < 0.001) meals whereas the comparison between POL and AHA were not significantly different (*P* > 0.05). **E**: Percent C18:2 significantly increased the most after AHA meal compared to PCAN (*a*, *P* = 0.037) and POL (*b*, *P* = 0.001) meals. The comparison between PCAN and POL was not significantly different (*P* > 0.05). **F**: Percent C18:3 significantly increased the most after PCAN meal compared to AHA (*P* > 0.05) and POL (*a*, *P* < 0.001) meals. The comparison between AHA and POL was also significantly different (*b*, *P* < 0.001). Legends **(A-F)**: POL = Palm olein only (); AHA = Soybean-Palm olein blend (); PCAN = Rapeseed-Palm olein blend ().

The effect of time was not significant after correction for the baseline values for individual fatty acids in both TRL and CE. Figures 3A-F show the distribution of individual fatty acids (mean ± SE) in TRL, expressed as a percentage of the FAC. A significant effect of dietary treatment (*P* < 0.05) was evident for palmitic (C16:0), oleic (C18:1), linoleic (C18:2) and linolenic (C18:3) acids in the composition of TRL but not for palmitoleic (C16:1n7) and stearic (C18:0) acids.

Incorporation of C16:0 in TRL was greatest after the POL test meal compared to either AHA and PCAN meals (*P* < 0.001) as expected due to its greater dietary availability (POL = 38.6%; AHA = 22.0%; PCAN = 13.5%) and this pattern was also reflected in its greater incorporation into TRL content after the AHA meal compared to the PCAN meal (*P* = 0.029). Percent C18:1 in TRL (*P* < 0.001) also reflected dietary availability (POL = 46.1%; AHA = 40.4%; PCAN = 55.2%) and incorporation appeared to be dose–dependent with PCAN > POL (*P* < 0.001), PCAN > AHA (*P* < 0.001) and POL = AHA (*P* > 0.05). Presence of C18:2 in TRL was significantly higher after the AHA treatment compared to the POL (*P =* 0.001) and PCAN (*P* = 0.046) treatments and this was dependent on the C18:2 content of the test meals (POL = 11.6%; AHA = 26.2%; PCAN = 18.1%). Treatment effects on C18:2 incorporation into TRL after PCAN and POL meals were not significantly different (*P* > 0.05). C18:3 was present in the AHA (3.7%) and PCAN (5.1%) meals only and TRL incorporation therefore reflected this dietary availability with both PCAN and AHA treatment effects significantly greater than POL (*P* < 0.001) treatment but not between themselves (*P* > 0.05). Of interest was the observation that these changes in plasma TRL FAC were already apparent when blood was sampled at 1.5 h post-meal challenge and continued with only negligible changes throughout the 7 h experimental duration.

**Figure 4:**
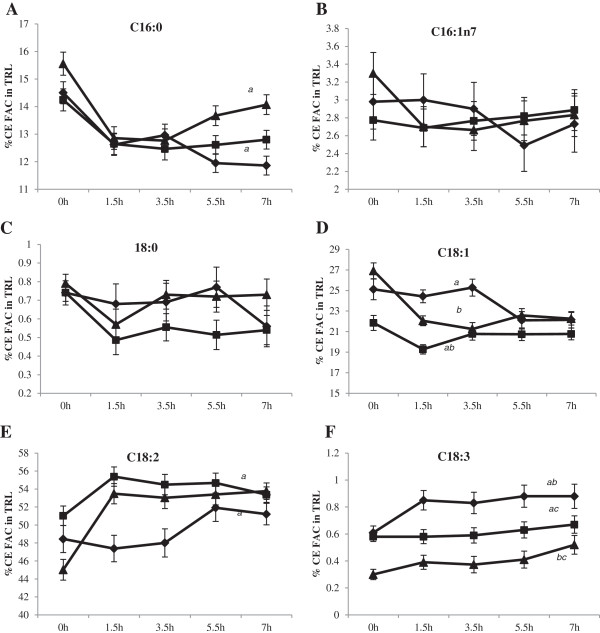
**Fatty acid composition (FAC) of plasma cholesteryl esters (CE) (mean % ± SEM, n = 15) in response to test meals containing POL, AHA or PCAN test fats. **^*2*^Statistical analyses were corrected for baseline values and significance reported at *P* < 0.05 for time x test meal interactions. **A**: Percent C16:0 in TRL CE significantly increased after the POL meal compared to the AHA meal (*a*, *P* = 0.043) but was not significantly different compared to the PCAN (*P* > 0.05) meal. The comparison between AHA and PCAN meals was not significantly different (*P* > 0.05). **B**: Percent C16:1n7 incorporation into TRL CE was not significantly different between meals (*P* > 0.05). **C**: Percent C18:0 incorporation into TRL CE was not significantly different between meals (*P* > 0.05). **D**: Percent C18:1 in TRL CE was significantly greater after POL (*a*, *P* = 0.002) and PCAN meals (*b*, *P* = 0.001) compared to the AHA meal whereas the comparison between POL and PCAN was not significantly different (*P* > 0.05). **E**: Percent C18:2 in TRL CE increased the most after the AHA meal compared to POL (*P* < 0.05) and PCAN (*a*, *P* = 0.017). The comparison between POL and PCAN was not significantly different (*P* > 0.05). **F**: Percent C18:3 incorporation into TRL CE was the most after the PCAN meal compared to AHA (*a*, *P* < 0.001) and POL (*b*, *P* < 0.001) meals whereas it was least after the POL meal. The comparison between POL and AHA meals was also significantly different (*c*, *P* < 0.001). Legends **(A-F)**: POL = Palm olein only (); AHA = Soybean-Palm olein blend (); PCAN = Rapeseed-Palm olein blend ().

Individual fatty acid concentrations in CE as percent FAC, in response to the varying P/S ratio test meals are presented in Figures 4A-F. Significant differences in CE fatty acids after correction for baseline values were evident for palmitic (C16:0, *P* < 0.05), oleic (C18:1, *P* = 0.017), linoleic (C18:2, *P* = 0.001) and linolenic (C18:3, *P* < 0.001) acids in the composition of TRL but not for palmitoleic (C16:1n7) and stearic (C18:0) acids (*P* > 0.05). The appearance of these fatty acids in CE reflected meal source but were independent of test meal × time interactions.

## Discussion

Fatty acid compositional analysis established chylomicron triacylglycerols (TAGs) closely followed the test fat FAC patterns for each dietary rotation in this study. Plasma TAG fatty acids mimic dietary fats and form a valid tool for compliancy measures [30]. The dietary fats in this study incorporated palm olein in varying proportions to achieve P/S ratios ranging from 0.27 (palm olein only), 1.0 Step 1 or AHA recommendation (palm olein with soyabean oil) which has a higher content of n-6 PUFA and 1.32 targeting a higher MUFA (palm olein + rapeseed oil) content which is the therapeutic lifestyle change (TLC) diet advocated by the ATP III guidelines. The overall fatty acid composition ratio of SFA:PUFA:MUFA achieved a proportion of 3.5:1:3.8 for the POL diet, 1:1.1:1.5 for the AHA diet and 1:1.3:3 for the PCAN diet. Palm oil in varying proportions allowed for the MUFA content to be kept constantly > 40% for all diets with only the proportion of palmitic acid as a SFA source and linoleic acid as a PUFA source differing between diets.

This study found that varying the P/S ratio did not significantly affect plasma TC and TAG levels postprandially. Plasma HDL-C concentrations were significantly affected by the P/S ratio of the diets tested. HDL-C concentrations increased with the POL diet (P/S = 0.27) but were lowered with the AHA (P/S = 1.0) and PCAN (P/S = 1.3) diets. A consequence of decreasing dietary fat saturation in humans is the concomitant decrease in HDL-C concentrations in both adult and pediatric populations [31-34]. In long-term studies it has been hypothesized that the lowering of HDL-C with increasing unsaturation was a consequence of isoenergetic substitution with carbohydrates [35-37]. We however kept carbohydrate content of all test diets constant, and the only dietary parameters that were interchanged were the SFA and *n*-6 PUFA content. Percentage increase in AUC plasma HDL-C for the POL diet compared to the PCAN diet was 31.4% whilst the increase for the AHA diet compared to the PCAN diet was only 8.4%. The increase in HDL-C caused by the POL diet during the postprandial period compared to the higher P/S diets may indicate an ability of the POL diet to promote reverse cholesterol transport (RCT). However, study limitations prevented the inclusion and evaluation of parameters such as HDL particle size or the enzymes involved in RCT such as cholesteryl ester transfer protein, lipoprotein lipase and lecithin:cholesteryl acyl transfer protein [38].

We observed a monophasic lipemic response irrespective of P/S ratios with peaking taking place between 3.5-5.5 h and the associated lipemia was not significantly different between test meals. However, the magnitude and duration of lipemia was greatest with the POL diet but lesser with oils of increasing P/S ratio. The monophasic pattern of postprandial TAG behaviors in this study is in variance with other studies which report peaking either once, twice or three times during the postprandial period [39-41]. A biphasic response has also been associated with increasing MUFA content after a single meal challenge [40]. In a study comparing palm oil, lard and puff pastry margarine, Jensen et al. reported a biphasic response curve with an initial peak 1-2 h and a second peak 4-7 h after the meal [40]. But the diet used was almost fat-free ~ 1 g of fat (total energy- 104 kcal; carbohydrates providing 83% energy; protein providing 10% energy). This was in stark contrast to the nutritional content of the postprandial meal supplied in this study (total energy 1010 kcal, 101 g carbohydrate, 53 g fat and 32 g protein) .

In agreement with our study, Pedersen et al. (1999) in comparing rapeseed oil, sunflower oil and palm oil as sources of MUFA, PUFA and SFA respectively, did not find significant differences between fat classes in relation to fat clearance and lipoprotein response [42]. Weintraub et al. (1988) also did not report any significant difference in TAG levels between SFA and *n*-6 PUFA diets [20]. However others have noted slower postprandial fat clearance of long-chain SFAs compared to *n*-6 PUFAs [19,21,43] whilst *n*-3 PUFA had the ability to markedly attenuate lipemia compared to SFA and *n*-6 PUFA diets [21]. Preferential hydrolysis by lipoprotein lipase for larger TRL particles has been reported in rat studies [44,45]. Therefore the lower lipemia caused by the AHA and PCAN diets compared to the POL diet may perhaps be explained by the particle size of TRLs which is increased by unsaturated fatty acids compared to SFAs [18,20].

The major finding of this postprandial human study was the post-meal effect of increasing HDL-C levels occurred with a decreasing P/S ratio which was achieved with a higher palmitic acid content. This finding is in agreement with other studies using palmitic-rich fats (palm oil) which suggest an association of palmitic acid with greater HDL-C levels and lower TC/HDL-C [46-48]. Increased HDL-C levels are cardioprotective with an anti-atherogenic benefit associated with its primary role in reverse cholesterol transport. A 1% increase in HDL-C results in a 1 to 2% reduction in major cardiovascular events [49]. Raising HDL-C is now a treatment goal for atherogenic dyslipidemia in CVD risk management in addition to reducing LDL-C [50,51]. This area of research is intense and remains complex as noted from the negative results associated with nicotinic acid or fibrate in combination therapy with statin [52]. The evidence from alternative efforts such as promoting exercise, moderate alcohol use, weight loss and smoking cessation as a means to promote HDL-C is scarce. Dietary factors that affect HDL-C remain to be identified [53].

## Conclusions

Lower P/S ratios of dietary fat blends using palm olein were associated with an increasing but non-significant lipemic trend in humans. On the other hand decreasing P/S ratios of these dietary fat blends were associated with a significantly greater prandial HDL-C trend. Although these data are important and interesting on their own merit, we recommend that that these effects should also be retested in trials with longer feeding periods and in subjects with more severe form of hyperlipidemia.

## Abbreviations

SFA: Saturated fatty acid; MUFA: Monounsaturated fatty acid; PUFA: Polyunsaturated fatty acid; TC: Total cholesterol; LDL-C: Low density lipoprotein-cholesterol; HDL-C: High density lipoprotein-cholesterol; TAG: Triacylglycerol; TRL: Triglyceride-rich lipoproteins; FAC: Fatty acid composition; POL: Palmitic acid-rich diet.

## Competing interests

Both authors have no competing interest. Dr. Sundram is employed by the Malaysian Palm Oil Council and uses science based facts and approaches to promote global palm oil use.

## Authors’ contributions

TK contributed dietetic supervision, laboratory management, data acquisition and drafting of this manuscript. KS made substantial contributions to conception and design of the study, statistical analysis and finalization of the manuscript. Both authors read and approved the final manuscript.
